# Micro‐Coil Neuromodulation at Single‐Cell and Circuit Levels for Inhibiting Natural Neuroactivity, Neutralizing Electric Neural Excitation, and Suppressing Seizures

**DOI:** 10.1002/advs.202416771

**Published:** 2025-04-17

**Authors:** Kayeon Kim, Xiyuan Liu, Bingdong Chang, Guanghui Li, Gwendoline A. E. Anand, Su Genelioglu, Alexandra Katherine Isis Yonza, Andrew J Whalen, Rune W Berg, Shelley I Fried, Anpan Han, Changsi Cai

**Affiliations:** ^1^ Department of Neuroscience Faculty of Health and Medical Science University of Copenhagen Copenhagen DK‐2200 Denmark; ^2^ Department of Civil and Mechanical Engineering Technical University of Denmark Lyngby 2800 Denmark; ^3^ Department of Neurosurgery Yale School of Medicine New Haven CT 06510 USA; ^4^ Boston VA Healthcare System Boston MA 02130 USA; ^5^ Department of Neurosurgery Massachusetts General Hospital and Harvard Medical School Boston MA USA

**Keywords:** MEMs micro‐coil, neural inhibition, neural interface, single‐cell study, two‐photon imaging

## Abstract

Micromagnetic stimulation (µMS) emerges as a complementary method for neuromodulation. Despite major advances in neural interface technology, there are limited options for neural inhibition. Here, a microchip‐based implantable micro‐coil device is presented to achieve high spatial precision for cortical inhibition. Cortical in vivo two‐photon imaging of spontaneous neural activity showed µMS reversibly suppressed single cells, and as µMS magnitude is increased, the suppressed cell population increased from 14% to 41%. At the circuit level, the average suppressed area is 0.05 mm^2^, seven times smaller than the activated area induced by micro‐electrode stimulation (µES). It is discovered that neurons responded more strongly to µMS than to µES, which is exploited to effectively neutralize the neural excitation induced by concurrently delivered strong µES (80 µA). Moreover, µMS mitigates hyperactive neural firing caused by pharmacologically induced seizures, reducing seizure amplitude by 54%. These findings underscore the potential of µMS as a precise, effective, and versatile tool for localized neuromodulation with an effect of opposite polarity from µES. Complementing optogenetic and electrical stimulation for multi‐functional neural interfaces, µMS holds promise as a unique neuroscience research tool and as a potential therapeutic intervention method for precisely suppressing hyperactive brain circuits.

## Graphical abstract

Micromagnetic stimulation (µMS) offers a precise method for neural inhibition, complementing existing neuromodulation techniques. Using an implantable micro‐coil device, µMS selectively suppresses cortical activity with high spatial resolution. It effectively counteracts micro‐electrode stimulation and reduces seizure amplitude, highlighting its potential for targeted neuromodulation and therapeutic applications in hyperactive brain circuits.

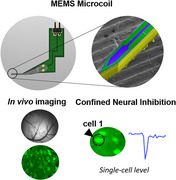

## 1 Introduction

The integration of chip technology to modulate brain activity through advanced neural interfaces (NIs) drives significant advancements in neuroscience and medicine. NI design involves the development of devices that directly interface with the nervous system^[^ ^1^, ^2^, ^3^, ^4^, ^5^, ^6^, ^7^ ^]^ to modulate functional or dysfunctional networks. Prominent clinical successes include cochlear implants, which enable speech recognition for the profoundly deaf,^[^ ^8^ ^]^ and deep brain stimulation (DBS), which alleviates symptoms in those with Parkinson's disease and other motor disorders.^[^ ^9^ ^]^ Beyond these, NIs are increasingly implemented to manage chronic and acute pain.^[^ ^10^ ^]^ Ongoing research continues to expand the potential of NIs,^[^ ^11^, ^12^, ^13^, ^14^, ^15^ ^]^ with promising developments in restoring vision to people who are blind^[^ ^16^, ^17^, ^18^ ^]^ and providing sensorimotor feedback to amputees.^[^ ^19^, ^20^ ^]^

Neurosensing via electrical sensors has proven to be a powerful tool for clinical applications, with recent research emphasizing the development of soft and biocompatible devices to improve integration with biological tissues.^[^ ^18^, ^21^, ^22^, ^23^, ^24^, ^25^, ^26^, ^27^ ^]^ While electrical sensing is a very powerful method, electrical stimulation has shortcomings, often resulting in over‐stimulation and undesired neuronal responses.^[^ ^28^, ^29^ ^]^ Optogenetics neuromodulation has emerged as an accurate and selective neurostimulation tool,^[^ ^30^, ^31^, ^32^, ^33^ ^]^ and it offers the unique ability to suppress specific populations of neurons with high precision. This makes optogenetics potentially beneficial for developing therapeutic interventions for conditions like epilepsy, where targeted inhibition of hyperactive neurons is critical.^[^ ^34^ ^]^ Other methods, such as transcranial magnetic stimulation (TMS), and invasive techniques like deep brain stimulation (DBS) have also shown suppressive effects on hyperexcitability.^[^ ^35^, ^36^ ^]^

Complementary to the above neurostimulation paradigms, micromagnetic stimulation (µMS) based on micro‐coils has emerged as an innovative neuromodulation method with unique advantages.^[^ ^37^, ^38^ ^]^ By inducing electric fields through micro‐coils, µMS enables precise modulation of brain activity without direct contact between the metal implant and surrounding neural tissue, enhancing safety and stability compared to standard electrodes.^[^ ^9^, ^38^, ^39^, ^40^ ^]^ While optical stimulation by our group^[^ ^41^, ^42^ ^]^ and others^[^ ^43^, ^44^ ^]^ is an established method for artificially targeting neural sub‐populations, and continues to evolve with improved biocompatibility, physical stimulation techniques like µMS are attractive because they provide a complementary and translational strategy, without the need for genetic modification. Furthermore, µMS offers focused activation, significantly improving spatial selectivity.^[^ ^45^, ^46^ ^]^ Similar to the use of magnetic stimulation at much larger spatial scales (e.g., TMS), µMS has demonstrated the potential for neural inhibition in brain slices or non‐vertebrate nerve systems,^[^ ^47^, ^48^, ^49^ ^]^ suggesting that it could be a powerful tool for managing hyperactive neuronal circuits. In this report, we explore this potential by exploring the following questions: Can µMS consistently and effectively suppress spontaneous neural firing or neurons in an actively firing state in living brains? How precise and localized are the effects? Can µMS reliably suppress hyperactive neurons under pathological conditions such as during seizures?

To answer the above questions and investigate the suppression effects of µMS with high spatial precision, we developed a novel MEMS‐based micro‐coil (MMC) system compatible with in vivo two‐photon microscopy (TPM). TPM is critical for examining cellular and subcellular responses with high spatial resolution in living brain tissue. However, previous micro‐coil designs were incompatible with this imaging modality, necessitating significant innovations in coil design. The newly developed MMC system includes several key advancements. The MMC was implanted in transgenic mice carrying cell‐type specific fluorescent indicators, and their stimulation effects were directly visualized. We chose the mouse visual cortex as a model for cortical processing because its well‐defined organization exemplifies system‐level principles shared across other sensory cortices, including motor and associative regions, somatosensory, barrel, and frontal cortices. Our approach yielded several novel discoveries, highlighting the exciting potential of µMS by MMC.

## 2 Results

### 2.1 Unique In‐Vivo Experiment Reveals Robust Focal Suppression by µMS

**Figure 1:**
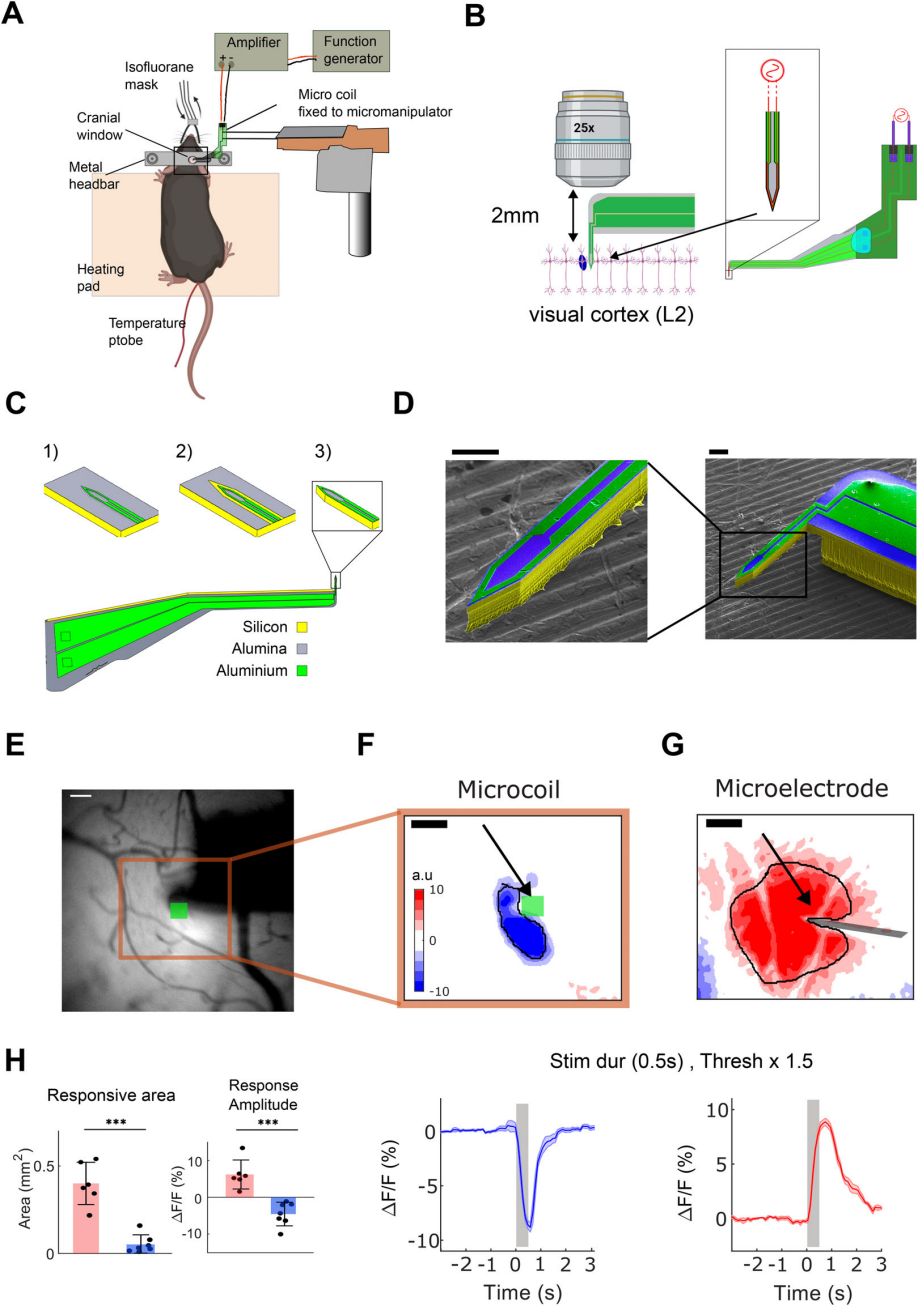
Overview of the experimental set‐up with dedicated MMC and µMS induced neuro‐suppression See text for details. (A) Schematic view of the experimental set‐up, and (B) the MMC penetrating the visual cortex layer 2 (L2) under the objective lens for two‐photon imaging. Inset; schematic view depicting current flow through the micro‐coil. (C) The MEMS fabrication process comprises: (C1) lithography and lift‐off patterned aluminum thin‐films, (C2) define the silicon probe geometry, (C3) release of the probe through plasma etching processes, which creates an 80‐µm‐thin implantable silicon probe. (D) Electron microscopy images of MMCs. Scale bar, 100 µm. (E) MMC in the visual cortex under the epifluorescence light. Green square indicates the vertically inserted micro‐coil, scale bar 80 µm. (F) Neuronal Ca2+ fluorescence (GCaMP8f) response to µMS. Scale bar, 150 µm. Lower panel: The response intensity time course, 5 stimulation trials are averaged, shaded with ± 1standard error mean (s.e.m.). (G) Same convention as (F), but response during µES. (H) Response area (left) and peak response amplitude (right) during micro‐coil (blue) and micro‐electrode (red) stimulation. Each data point represents a mouse tested under medium‐level stimulation conditions. *p < 0.01, **p < 0.001, ***p < 0.0001.

A schematic of the experimental set‐up is shown in **Figure** **1**. The delivery of stimulation from the MMC (or micro‐electrode) is synchronized with the recording of neural activity during in‐vivo mouse neuroscience experiments. The cutting‐edge system includes a TPM, wide‐field imaging for capturing both micro and macro‐level neural responses^[^ ^50^ ^]^ (Figure 1A). Microsurgery is performed to allow the visual cortex to be imaged through an optically accessible cranial window (see Materials for details.). The MMC, fixed to a micromanipulator, is inserted into the visual cortex to a depth of 200 µm from the cortical surface (corresponding to Layer 2/3, Figure 1B). A function generator is connected through a custom amplifier to the MMC, which allows us to pass time‐varying current through the MMC, thereby inducing an electric field, that if strong enough, can modulate activity in surrounding neurons.^[^ ^51^ ^]^ We custom‐made a new MMC, because existing micro‐coil designs did not meet the requirements for this study (including MMCs^[^ ^46^, ^51^ ^]^ made previously by our group). Briefly, the goals for the new MMC device are: 1) to have a thin cross‐sectional profile that minimizes brain damage upon insertion and maximizes the visualization of neurons during two‐photon imaging; 2) compatibility with the 2 mm working distance of the TPM high magnification objectives needed to study single‐cell responses with MMC inserted; 3) enable insertion of the probe at a perpendicular orientation to the cortical surface, enabling the spatial spread of activation to be better confined.^[^ ^51^ ^]^ To achieve these objectives, the fabrication of these “pick‐arms”, like MMCs, consisted of three main stages. In stage 1 (Figure 1C1), an insulating alumina layer was deposited on both sides of a silicon wafer. Lithography and lift‐off patterned aluminium thin films to conduct the electrical current. A second alumina layer is deposited as an electrical insulation layer (not shown to increase clarity). Patterning of the alumina layer through plasma etching^[^ ^52^, ^53^ ^]^ defines the silicon probe geometry in the plane of the silicon wafer and exposes the aluminum pads needed for wire‐bonding (Figure 1C2). A 2‐step DRIE plasma etching process creates an 80‐µm‐thin implantable silicon probe and frees the MEMS device from the wafer^[^ ^54^ ^]^ (Figure 1C3).

The MMC was glued to a custom PCB with gold‐plated contacts and wire‐bonding used to connect the probe to the PCB. The assembled device was encapsulated with a parylene C coating, which completely electrically insulated the micro‐coil device, and helped to increase implant biocompatibility. The MMC had an 800‐µm‐long, 80‐µm‐wide, 80‐µm‐thick, needle that is perpendicularly inserted into the mouse cortex (Figure 1B,E). The current carrying aluminum wire is 2‐µm‐thick and 10‐µm‐wide (Figure 1B, right inset).

We utilized wide‐field imaging to capture macroscale neural activity while ensuring the safe insertion of the stimulation probes. The narrow profile of the MMC helped to avoid blood vessels during insertion of the probe as well as to minimize tissue‐damage (Figure 1E). In the experiments, we utilized mice that expressed the genetically encoded green fluorescent calcium indicator (GCaMP8f).^[^ ^55^ ^]^ This allowed the activity of cortical neurons in an area larger than 3 × 3 mm^2^ in and around V1 to be visualized with high temporal resolution (Figure 1E).

In preliminary experiments, we consistently observed robust neural suppression during µMS (Figure 1F). This observation was not due to an anomaly in our experimental system as micro‐electrode stimulation (µES) similarly produced neural excitation (Figure 1G). The suppression effect was consistent across multiple stimulation trials, animals, and micro‐coil devices (see below). In addition to the polarity of the response, the area affected by µMS was significantly smaller than that from µES responses. This occurred even though the µES probe tip was very small (radius of 2 µm, which is much smaller than the MMC probe), the µMS activation area remained significantly more localized compared to µES. Specifically, the activation area of µMS was seven times smaller than that of µES (Figure 1H, µMS, 0.052 ± 0.05 mm^2^; µES, 0.4 ± 0.12 mm^2^).

This difference in the size of the responsive area agrees with finite element method simulations (FEM) that compared the electrical field strengths of the two stimulation methods. For the micro‐electrode, high field strength surrounded the entire metallic wire (Figure S1, Supporting Information). In contrast, for µMS, high field strength was confined near the edge of the MMC tip. FEM simulations of the field gradient in the z‐direction (dEz/dz) are shown in Figure S1A,B (Supporting Information). The peak field gradient for the micro‐coil exceeded 11 000 V m^−2^, surpassing the threshold for effective magnetic stimulation,^[^ ^51^ ^]^ as reported for peripheral neurons in response to stimulation from a large TMS coil. The smaller activation volumes and higher spatial accuracy observed in the numerical simulations further support the high spatial precision of MMC stimulation compared to µES, observed in physiological experiments µMS.

### 2.2 µMS Suppresses Neuronal Activities at the Single‐Cell and Population Levels

**Figure 2:**
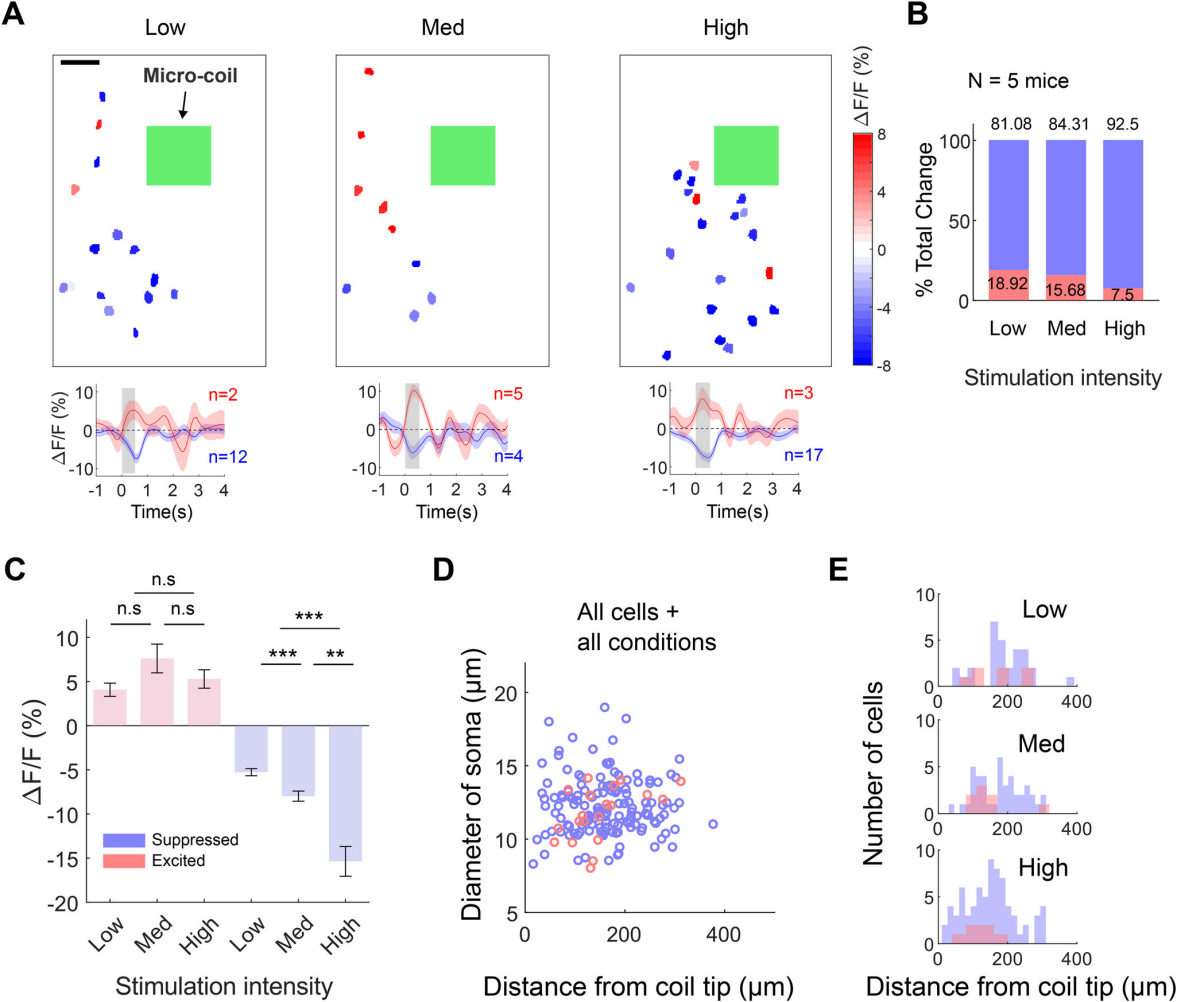
TPM imaged neuronal activity during µMS. (A). The somatic response amplitude cascade (1s‐post stimulation) by low, medium, and high stimulation intensity, scale bar: 50µm. Green square: micro‐coil cross‐section. Lower panels show corresponding response traces averaged across excited (red) and suppressed cells (blue). Shaded gray line with ±1s.e.m., indicates stimulation duration. (B) % total change of excited (red) and suppressed (blue) is plotted against stimulation intensity. (C) Ca^2+^ response amplitude (ΔF/F %) during the post‐stimulation 1s period across all cells that are significantly excited (red) and suppressed (blue) is plotted against stimulation intensity. Error bars represent 1s.e.m. (D) The diameter of soma is plotted against the location of individual cells recorded relative to the micro‐coil center. (E) Same convention as (D), but individual cells are plotted into low (upper), medium (middle), and high (lower) stimulation intensities. Y‐axis, distance from the micro‐coil location. Red, excited cells; blue, suppressed cells. *p < 0.01, **p < 0.001, ***p < 0.0001.

Measurement of responses in single neurons with TPM revealed that most were suppressed (*n* = 12/14) during µMS (**Figure** **2**A). As the stimulation intensity was increased (see Experimental Section), there was a noticeable increase in the density of suppressed cells (Figure 2A,B). Looking across the population of cells that were sensitive to µMS (30, 43, 74 cells for low, medium, high intensities, respectively, from 5 mice), a larger proportion were significantly suppressed (Figure 2B), e.g., the percentage of suppressed cells increased with stimulation intensity from 14% at low to 23% at medium, and 41% at high intensities. In contrast, µES showed the opposite effect, i.e., the majority increased while only a subset of cells was suppressed (Figure S2, Supporting Information). While only 3–4% of neurons were excited by low levels of µMS, increasing the intensity did not increase the proportion of excited cells. Furthermore, with higher stimulation intensity, the strength of the response in suppressed neurons was even greater. In contrast, the responses of excited neurons remained unchanged (Figure 2C). Taken together, these results indicate that most nearby neurons are suppressed by µMS, and furthermore, the magnitude of suppression can be systematically modulated by adjusting µMS intensity.

To explore whether µMS preferentially modulated a specific population of cortical neurons, we plotted the diameter of each neuron that responded to µMS as a function of its distance from the implanted MMC and generated separate plots for excitatory and inhibitory cells (excitatory pyramidal neurons are known to have larger somas than inhibitory interneurons^[^ ^56^ ^]^). There was no difference in soma diameter between significantly suppressed or excited cells, as well as in relation to the distance from the electrode location (Figure 2D). Also, the distribution of excited cells and suppressed cells did not differ in intensity (Figure 2E), indicating that µMS suppressed both excitatory and inhibitory neurons.

### 2.3 µMS‐Induced Suppression Elicits Faster Neuronal Responses Compared to µES

**Figure 3:**
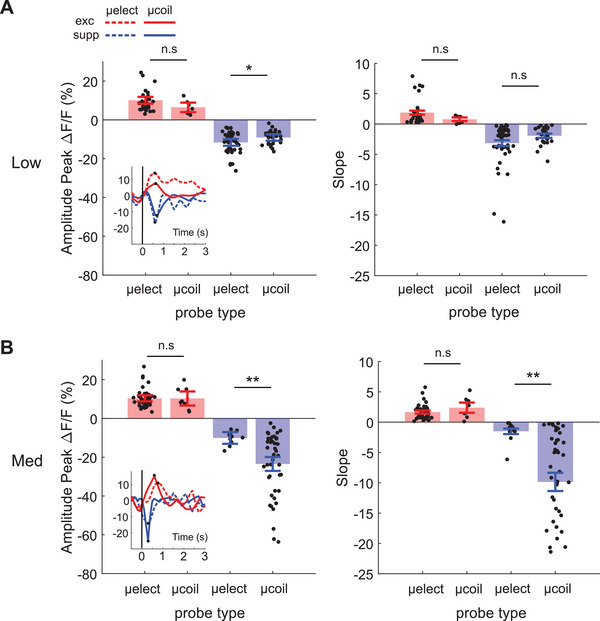
Somatic response comparison during µES versus µMS from TPM. (A). Response amplitude peak (left), and slope (right) of individual cells that are excited (red) and suppressed (blue) are plotted during micro‐electrode and micro‐coil low amplitude intensity. The small inset shows example response traces during µES (dashed line) and µMS (solid line), with a vertical line indicating stimulation onset. Small dots indicate response peak of each trace. (B). Same convention as A but plotted response amplitude peak and slope during medium stimulation intensity. *p < 0.05, **p < 0.005.

TPM enables responses to be observed not only at the cellular level but also from subcellular structures, i.e., somatic and neuropil responses can be differentiated. This distinction is particularly valuable, as a sharp increase in calcium levels within the soma indicates neuronal firing.^[^ ^57^ ^]^ **Figure** **3** shows the pooled somatic responses of individual neurons from all mice and stimulation sites that showed significant modulation in response to stimulation. There was no significant difference in the peak amplitude or slope of the response when µMS and µES were compared (Figure 3). Note however, that more neurons were activated during µES than µMS, as µES activates larger areas.

Interestingly, when stimulation intensity was increased, it was not the excited cells, but rather the µMS‐suppressed cells, that exhibited significantly larger negative peak amplitudes and steeper slopes (Figure 3B). These findings suggest that µMS‐induced suppression is associated with stronger and more rapidly changing deflection in the calcium signal, reflecting a more pronounced and dynamic suppression of neuronal activity. Remarkably, neuronal responses under µMS were stronger and faster than the responses to µES. The ability to generate fast‐acting suppressive signals is intriguing, as it raises the possibility that certain types of excitation, such as the unwanted hyperexcitability associated with some diseases can be mitigated. This is explored further in a later section.

### 2.4 Neuropil Responses Reveal Confined Response by µMS

Neuropil refers to the network of dendrites, axons, and synapses surrounding the neuron's cell body (soma). Compared to somatic activity, neuropil measurement requires minimal signal processing while serving as a reliable indicator of collective neural activity enabling a clear view of activity spread.^[^ ^58^ ^]^ This signal can further be utilized in both pre‐clinical and clinical closed‐loop brain‐computer interface (BCI) applications.^[^ ^59^, ^60^ ^]^ Inspired by these advantages and to explore how this signal contributes to network‐level responses observed in our initial wide‐field imaging investigation, we extracted neuropil activity from TPM recordings by masking out all neuronal somata.

**Figure 4:**
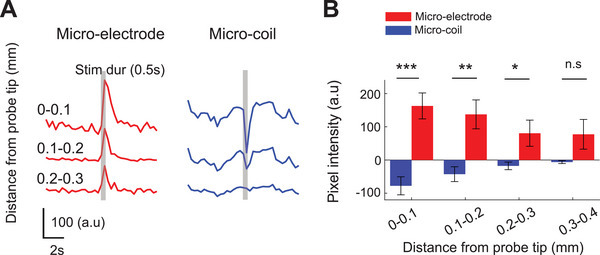
Neuropil response comparison between µES and µMS. (A) Example traces of neuropil response time course with segments of 0.1 mm from the probe tip during µES (red) and µMS (blue). Gray bars indicate stimulation duration. (B). Bar plot with pixel intensity comparing µES (red) and µMS (blue). *p < 0.05, **p < 0.005, ***p < 0.0005.

Consistent with our wide‐field imaging results (Figure 1E–H), we found that suppression during µMS was most pronounced around the micro‐coil, analogous to stronger excitation from µES near the electrode. During µMS, the neuropil response attenuated within 200 µm of the micro‐coil tip in some populations of cells (**Figure** **4**A) and was mostly eliminated within 300 µm across all experiments (Figure 4B). In contrast, activation from electric stimulation was still prominent at distances >400 µm.

Using the more spatially precise information available from TPM, these findings confirmed that µMS induces localized suppression around the micro‐coil tip, in contrast to the broader excitatory effects of µES. This also validates our observations from wide‐field imaging and quantitatively confirms the spatial extent of stimulation effects under our specific experiment conditions.

### 2.5 µMS for Neutralization of Micro‐Electrode Neural Excitation and Cortical Seizure Suppression

**Figure 5:**
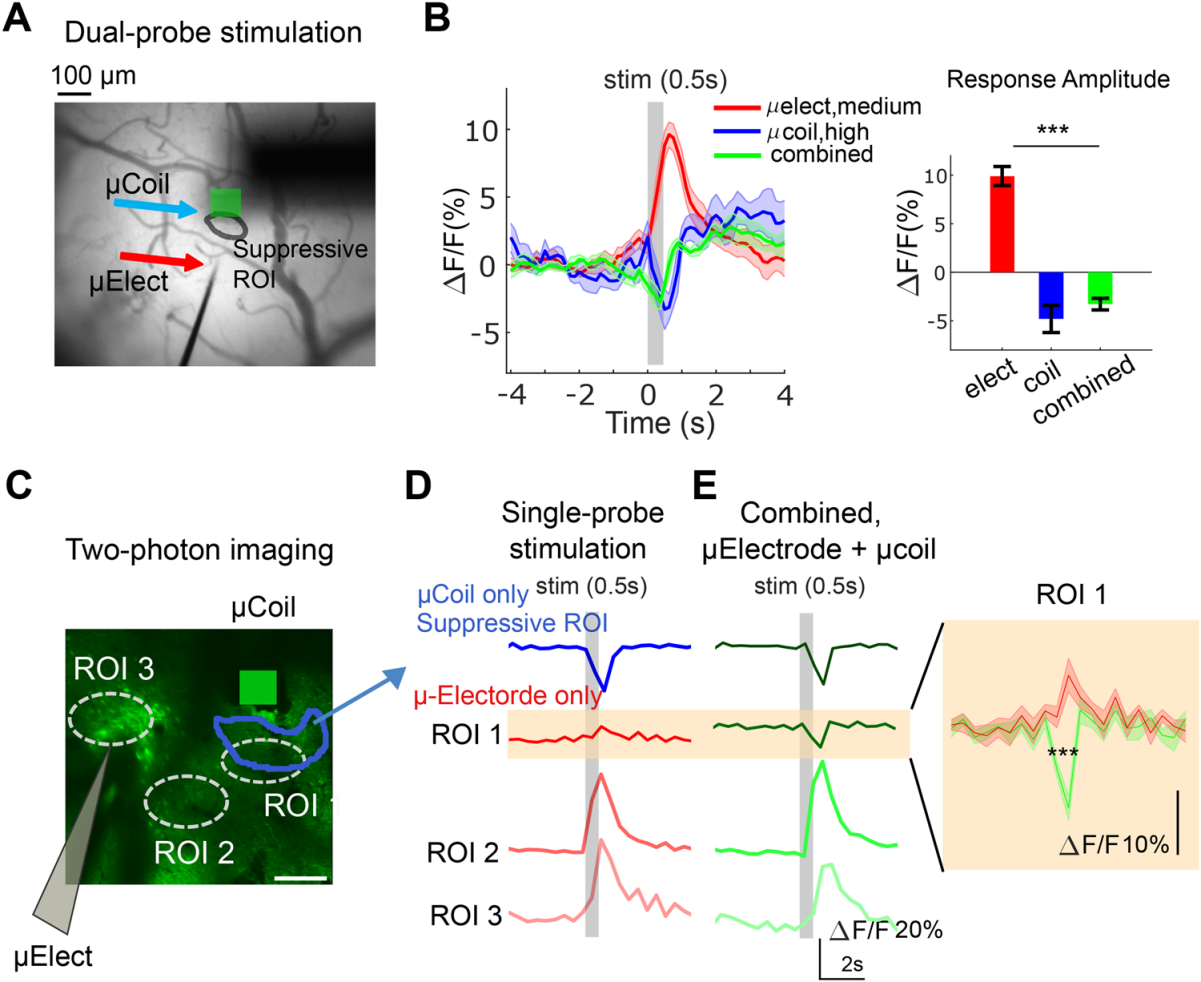
µMS neutralized µES. (A) Dual‐probe stimulation was set up using a micro‐electrode and MMC under wide‐field imaging. The green bar indicates a cross‐section of the micro‐coil (B) Left, time‐course of neuronal activity within the suppressed ROI (black contour shown in A). Responses to µES only (red), µMS only (blue), and simultaneous stimulation of both probes (green). Shaded areas indicate ±1 s.e.m. across five repeated stimulation trials. Right, bar plot showing peak amplitude (ΔF/F%) across the three same conditions. (C) Dual‐probe stimulation under TPM. The white dashed circle indicates the ROIs that outline response traces in panels D and E. The blue contour shows the region significantly suppressed by µMS only. Scale bar, 100 µm (D) Response amplitude (ΔF) within each ROI indicated by the white dashed circle in C during single‐probe stimulation; µES (red), and µMS (blue) stimulation. (E) Same convention as in (D), but showing the response to synchronized stimulation. Inset: expanded view of response waveforms of ROI 1, red, electrode only; green, combined condition. ***p < 0.0001.

Our findings that somatic responses to µMS are stronger and faster than µES (Figure 3), raise the intriguing possibility that µMS could suppress or even neutralize excitatory responses such as those triggered by µES. **Figure** **5**A shows wide‐field images of an MMC implanted 200 µm away from a micro‐electrode. This enables µES to activate areas in V1 that are close to the implanted MMC. Intriguingly, the suppression arising from µMS reduced the activation triggered by uES in the ROI nearest to the MMC. Within the primary suppression zone (Figure 5A, black contour, B, blue trace), neural responses to electrical activation were completely neutralized by MMC.

The electrical activation could not overcome the µMS suppression (Figure 5B). The two‐photon imaging results also agreed with the wide‐field imaging result, showing the excitatory responses by µES were neutralized during concurrent µMS. We examined the data by defining three ROIs and captured their response to stimulation from the micro‐electrode (Figure 5D, left). We then repeated the experiment while delivering µMS simultaneously with µES (Figure 5E). A significant suppressive effect was observed near the micro‐coil (Figure 5D, E, ROI 1). Thus, the wide‐field and two‐photon experiments support the notion that µMS can neutralize micro‐electrode neuroactivation, at least under certain conditions.

**Figure 6:**
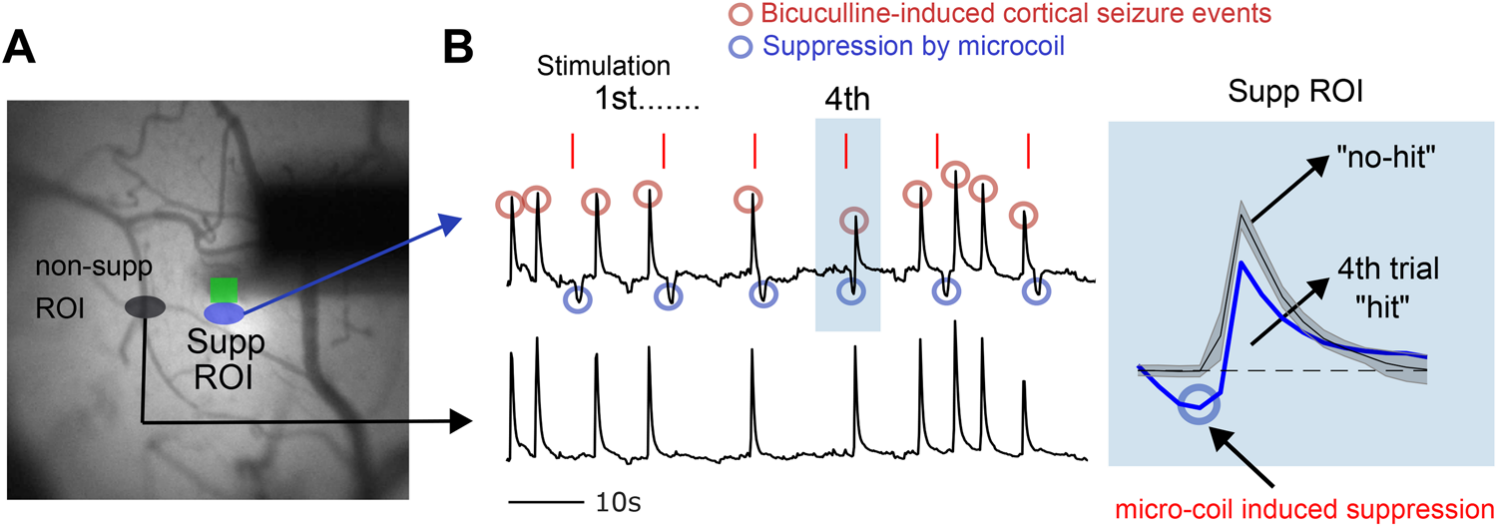
Inducing cortical seizure events to test the efficacy of µMS during the pathological state of the cortex. (A) The blue circle is the locally suppressed area (supp ROI). The black circle is a non‐suppressive area (non‐supp ROI). B) Upper trace: Neuronal response within suppressive ROI induced by µMS (blue open circles) during cortical seizure events (red open circles). Lower trace: response trace for non‐suppressive area. Red vertical lines indicate µMS stimulation trials. Right inset: superimposed waveforms within the suppressed ROI, “hit” at the fourth stimulation trial (blue) versus “no‐hit” waveforms (averaged, black). Shading indicates ± 2 s.e.m.

To further investigate the potential of these results for clinical interventions, we explored the use of µMS suppression for reducing the hyperactivation arising during pharmacologically induced seizures. We topically applied high concentrations of bicuculline (100 µm) on V1, especially the exposed region of cortex within the cranial window and implanted a MMC (**Figure** **6**). The bicuculline effectively induced strong bursts of activity over the entire imaging area; bursts occurred every 5–20 s. Stimulation was then delivered to the implanted MMC, and we compared the strength of the burst responses with and without µMS. In the more distal ROI (Figure 6A, black oval), there were periods of robust hyperactivity following the application of bicuculline, with little evidence of suppression from µMS. In the ROI closer to the MMC however (blue oval), there were clear periods of transient suppression triggered from µMS. Interestingly, when suppression arrived immediately prior to the burst of activity, there was a substantial reduction in its magnitude. The peak magnitude in this case was reduced by 54% compared to the largest unaffected waveform, exhibiting the smallest peak amplitude across all other unaffected waveforms (Figure 6B, showing the fourth trial of µMS stimulation, with the right blue box showing superimposed signals averaged across “no‐hit” waveforms).

The fact that µMS can focally suppress and possibly even neutralize the neuronal hyperactivity is intriguing because it raises the possibility that small arrays of implantable MMCs might one day be useful as a means to reduce seizures or other adverse effects associated with neuronal hyperactivity.

## 3 Discussion

For the first time, we reported a new experimental set‐up to resolve central questions about the suppressive effect of µMS. This platform allowed us to comprehensively examine neuronal responses at both single‐cell and population levels, enabling direct comparisons between µMS and µES. µMS induced robust and reversible suppression, as the neurons and area affected by uES consistently returned to their resting state, enabling repetitive stimulation. Further, µMS produced spatially confined neuromodulation, ≈7 times smaller than µES. Achieving a remarkable and surprising discovery, we observed that neurons reacted more strongly and sharply to µMS, and systematic modulation was viable with varying stimulation intensities. Exploiting this extraordinary observation, we then explored applications of µMS‐induced suppression and neutralization in two scenarios. First, combining simultaneous µMS and electrical stimulation exhibited a canceling effect where µMS effectively neutralized neural excitation elicited by concurrent electrical stimulation. Second, in a pharmacologically induced seizure model, we demonstrated that µMS could effectively suppress hyperactive neural firing in the area near the implant. Together, these findings highlight µMS for robust neural modulation and suggest promising applications in controlling NIs based on electrical stimulation and managing hyperactivity‐related brain disorders.

### 3.1 Neuro‐Suppression by MMC

Based on the clinical success of TMS suppression, our previous in‐vitro^[^ ^47^ ^]^ and non‐vertebrate µMS studies by others^[^ ^48^, ^49^ ^]^ hypothesized the potential for the arrest of epileptiform activity. While in vitro brain slices are a powerful experimental model due to their accessibility and manipulability, it is unclear just how well the results translate to in vivo conditions,^[^ ^61^ ^]^ primarily because they lack complex interactions among different cell types. Additionally, stimulation experiments on brain slices typically allow recording a small number of cells at a time, making it difficult to observe effects at the population or network level. Moreover, previous studies have not reported the suppressed area's size nor addressed the consistency and reliability of the suppressive effect in intact living brains. Our in‐vivo findings address these gaps by demonstrating that the majority of neuronal somata recorded under TPM were immediately suppressed upon µMS stimulation. Beyond single‐cell responses, our wide‐field imaging reveals for the first time that µMS‐induced suppression occurs within the local microcircuitry at the stimulation site, effectively reflecting the responses of subcellular structures, including neuropil. This finding on the local network effect is particularly interesting. While previous µMS studies suggested confined effects,^[^ ^45^, ^46^ ^]^ they lack precise quantification. Here, we clarify and quantified that the effective suppression area is 0.052 ± 0.05 mm^2^, and those single cells responded more strongly and rapidly with higher stimulation intensities. Care must be applied with these values however, given that the optimal interaction volume depends on the therapeutic goal. While broader stimulation may be beneficial in some cases, a smaller interaction volume will sometimes be essential for precise modulation of specific neural substrates. Localized neurostimulation may lead to unintended rebound of neural activities,^[^ ^62^, ^63^ ^]^ and local inhibition could lead to network disinhibition,^[^ ^64^ ^]^ where inhibiting a focal area weakens surrounding inhibitory control. The extent of these physiological and network‐level effects, whether beneficial or detrimental, may vary with stimulation parameters,^[^ ^65^, ^66^ ^]^ highlighting the importance of carefully tuning the interaction volume to balance precision and efficacy. Building on the clinical success of TMS and addressing the limitations of broader DBS approaches,^[^ ^67^, ^68^ ^]^ focused neuroinhibition with MMC has the potential to enhance treatment precision for conditions such as focal epilepsy, where it may help arrest seizures before they spread to a larger network,^[^ ^63^ ^]^ as well as Parkinson's disease and dystonia, where modulation is thought to target only a small neural circuit. Our findings demonstrate the efficacy of µMS in suppressing seizure‐related neural activity, serving as proof of concept for this approach. By directly targeting pathological activity at its source – such as epileptic foci^[^ ^69^ ^]^ or hyperactive microcircuits in thalamus^[^ ^70^ ^]^ µMS holds promise for improving therapeutic outcomes while minimizing unintended neural disruption.

### 3.2 µMS by MMC as a Novel and Versatile Neuro‐Suppression Tool for Neuroscience

Our results highlight the potential of µMS as a new tool for reversible, accurate, and reliable suppression of hyperactive neurons. One of the advantages of µMS as a neuro‐suppression technology is its versatility in target areas for implantation and stimulation. The versatility of µMS by MMC enhances its potential to serve as a multi‐functional neuromodulation platform or become an additional modality for multi‐functional probes^[^ ^71^, ^72^, ^73^, ^74^ ^]^ such as the Utah micro‐electrode Array,^[^ ^75^ ^]^ and flexible and soft brain‐machine interfaces.^[^ ^11^, ^76^ ^]^ For example, integrating complementary micro‐electrodes for neuro‐excitation and recording with MMC for neuro‐suppression could enable directional neural modulation. This approach would neutralize excessively stimulated areas while maintaining neural excitation as the primary goal of the application. For the Utah micro‐electrode arrays, stimulation currents are smaller than 100 µA, a level that our results here suggest that MMCs could effectively cancel. This bidirectional modulation tool can also potentially serve as a powerful method for gaining a deeper understanding of neural microcircuit mechanisms. For example, within the hippocampal microcircuit and its reciprocal connections with the entorhinal cortex,^[^ ^77^ ^]^ targeted disruption or suppression of specific circuit relays can be used to examine the input‐output dynamics of these circuits, providing insights into their functional relevance to memory and learning.^[^ ^78^ ^]^

Optogenetic neuro‐suppression and infrared neural stimulation (IINS)^[^ ^79^ ^]^ are well‐established technologies for directly inhibiting neural activity. IINS utilizes infrared light to induce transient tissue heating, which can activate or suppress neurons for the regions being targeted.^[^ ^80^, ^81^, ^82^ ^]^ While optogenetics requires gene modification, IINS does not; however, both methods are influenced by light scattering and often require physical tethering.^[^ ^24^, ^83^, ^84^ ^]^ As a complementary approach, µMS has its own strength including targeted neuromodulation through direct electromagnetic stimulation without genetic engineering. Further, although the recent data suggested that micro‐coils in other studies induce temperature rises ≈1 °C, this is thought to be within the acceptable range of biosafety^[^ ^85^ ^]^ and clinical implantation.^[^ ^83^, ^86^ ^]^ Thus, µMS could potentially complement optogenetic and IINS for direct neuro‐suppression for treating different illnesses caused by undesired neuronal activity such as epilepsy.

## 4 Conclusion

We demonstrated the versatility of µMS across various neural activity states. Specifically, µMS was shown to 1) suppress spontaneous neural firing, 2) neutralize neuronal excitation induced by µES, and 3) reduce epileptiform seizure activity. In light of these advantages, µMS potentially complements optogenetic and IINS for direct neuro‐suppression for treating conditions caused by undesired neuronal activity, such as chronic pain,^[^ ^10^ ^]^ epilepsy,^[^ ^34^ ^]^ tremors, and perhaps even abnormal appetite that leads to severe obesity.^[^ ^87^ ^]^

## 5 Experimental Section

### Animals

Throughout the experiment, >8‐week‐old C57BL/6 mice (*N* = 17, including both females and males) with a weight range of 20–35 g were used. For µMS, the sample size was *N* = 10 (five mice for two‐photon imaging, five mice for wide‐field imaging, with three mice shared between both imaging modalities). For electrical stimulation, the sample size was *N* = 9 (four mice for two‐photon, six mice for wide‐field, with one mouse shared between both imaging modalities). Within the 17‐mouse cohort, 2 mice were subjected to simultaneous stimulation of micro‐electrode and micro‐coil stimulation and pharmacological control. Some mice contribute to two data points for wide‐field imaging (micro‐electrode *N* = 6 mice, *n* = 6 stimulations, 5–10 repetitive trials per stimulation; micro‐coil *N* = 5 mice, *n* = 7 stimulations) for population results. The research was conducted in compliance with the guidelines outlined in Directive 2010/63/EU of the European Parliament and the Council regarding the care and use of animals for research purposes. All procedures were followed and approved by the Danish National Committee on Health Research following the European Council's Convention for the Protection of Vertebrate Animals used for experimental and other scientific purposes.

### Fabrication Process of MEMS Micro‐Coil in Brief

The MEMS fabrication process comprises 3 stages, 15 steps, and incorporates 4 lithography masks (Figure 1A,B). This new process yields much more accurate devices than our previous work.^[^ ^46^ ^]^ In the initial stage, both sides of the Si wafer were coated with a 100‐nm‐thick aluminium oxide layer using atomic layer deposition (ALD). The aluminium micro‐coil's patterning was realized via “lift‐off.” Subsequently, an Al thin film was deposited through physical vapor deposition (PVD). In the second stage, the process involves five crucial steps for patterning alumina. Initially, an ALD alumina layer, precisely 100 nm thick, was deposited on the Si wafer to electrically insulate the Al wire. The following steps utilize a combination of photolithography and plasma etching to pattern the alumina layer on the wafer's front side. After completing the above steps, the Al wire's bonding pads, as well as the Si on the back side of the wafer, become exposed. In the third stage, the surrounding silicon was etched using DRIE to create a height difference of 70 µm between the probe and the substrate. The backside Si was then reduced to a thickness of 20 µm via DRIE, resulting in an 80‐µm‐thick silicon cantilever after the DRIE etch. Subsequently, the remaining silicon was etched through.

The MEMS device packaging procedure encompasses four steps: first, the probes adhered to a custom PCB with gold‐plated contacts necessary for wire bonding. Second, the probes underwent ball wire bonding utilizing gold wire. In the third step, the wires were sealed with epoxy glue. Lastly, the fully assembled device received a coating of 6‐µm‐thick parylene C.

### Experimental Procedures—Viral Vector Injection

Two weeks before the recordings, the animals (*N* = 17) received injections of Adeno‐associated viral vector (AAV) carrying the neuronal‐specific calcium indicator GCaMP8f (pGP‐AAV‐syn‐jGCAMP8f‐WPRE; Addgene #162376‐AAV9) at three different depths (200 nl for each depth) targeting the visual cortex (+1 mm AP, + 2 mm ML relative to lambda).

### Experimental Procedures—Animal Preparation

During the acute experiment, lidocaine (10 mg kg^−1^) was administered for local anesthesia before making a surgical incision. After the craniotomy, the dura was peeled off. Following probe insertion, agarose was applied to stabilize both the cortical surface and the probe fixation. The body temperature was maintained at 37 °C throughout the procedure using a heating pad. Isoflurane was used for anesthesia induction at a concentration of 4% and was maintained at 0.9–1.5% throughout the experiment. Pharmacologically, bicuculline (100 µ_M_, Tocris Bioscience) was applied topically to induce cortical seizures.

### Stimulators and Stimulation Protocol—Micro‐Coil Magnetic Stimulation

During µMS, a function generator (Agilent 33250A) connected to an audio amplifier was used for signal output (Figure 1A). A 1 kHz sinusoid wave that was −90⁰ phase shifted with a pulse duration of 1 ms, and a stimulation delivery frequency of 200 Hz were used (Figure 1B).^[^ ^45^ ^]^ Given the variability in micro‐coil properties, such as impedance across custom‐built batches (0.8–2.7 Ω), intensity thresholds were established through initial responses observed in wide‐field imaging. Initially, “low” intensity was defined as the response to threshold‐level stimulation, typically ranging from 90 mV (input voltage of function generator) to 130 mV. Subsequently, the intensity was increased to “medium,” corresponding to an intensity increase within the range of 10–40 mV from the low intensity. Finally, “high” intensity was defined as an increase within the range of 20–50 mV from the medium level.

Throughout the experiments, input amplitudes ranging from 90 mV (lowest) to 220 mV (highest) were used, corresponding to output voltages of 1–3 V through the amplifier, which translated to current ranges of 50–110 mA. Variable stimulation durations were employed, with an average of 0.46 ± 0.15 s, ranging between 0.2 and 0.6 s. During the dual‐probe and seizure‐induced experiments, intensity was categorized as medium current for electrical stimulation and high intensity from µMS, ensuring the intensity was high enough to elicit excitation from the micro‐electrode and suppression from micro‐coil. This threshold‐based stimulation current approach allowed us to achieve comparable response amplitudes during micro‐coil and micro‐electrode stimulation despite the distinct parameters of the two devices.

### Stimulators and Stimulation Protocol—Electrical Stimulation

For electrical stimulation, ISO‐flex (A.M.P.I.) was used connected to Platinum‐Iridium micro‐electrodes (8‐11kΩ, tip diameter, 2–3 µm; PI2PT30.01. A3; Microprobes for Life Sciences). The reference electrode was connected under the neck skin of the mouse. The electrode was secured to the arm of a micromanipulator (Figure 1F) with an insertion angle of 15–20° for two‐photon imaging and inserted 200 µm from the surface of the cortex as the stimulation depth. A cathodic‐leading biphasic pulse (200 µs per phase, no interleaving between pulses) was used with a stimulus duration varied between 100 and 900 ms to compensate for the sampling frequency during imaging acquisition. The stimulation frequency was fixed at 200 Hz across all recording sessions and all subjects.

Similar to µMS, wide‐field imaging was used to establish the response threshold (Figure 1G), starting from a very low current of ≈4 µA. Then, stimulation intensity was increased in steps of 2–5 µA^[^ ^88^ ^]^ until observing the calcium signal increase in response to stimulation. Subsequently, the stimulation intensity was set between 1.2 and 1.5 times the response threshold during experiments. The average stimulation intensity during imaging was 21.8 ± 4.3 µA for wide‐field (mean ± standard deviation, *n* = 6, range: 18–30 µA, average stimulation duration: 0.2 ± 0.24 s) and 17± 6.27 µA for two‐photon (*n* = 4, range: 10–25 µA, stimulation duration: 0.65 ± 0.19 s). During two‐photon imaging, the stimulation intensity was increased from a low level (threshold level) to a medium level, using increments of 2–5 µA. This procedure was conducted in two mice to compare the effects with the medium‐level stimulation intensity of µMS. A total of 5–10 stimulation trials were performed with a 10‐s inter‐trial interval following threshold determination.

### Two‐Photon and Wide‐Field Imaging

A two‐photon microscope (FluoView FVMPE‐RS, Olympus) equipped with a femtosecond laser (Mai‐Tai DeepSee) and 25 × 1.05 NA water‐immersion objective, along with GaAsP detectors were utilized. For neuronal GCaMP8f recordings, an excitation wavelength between 850 and 920 nm was used. Image acquisition frequencies ranged from 1.81 to 4.38 Hz, adjusted according to pixel density and field of view. For wide‐field imaging, Olympus U‐HGLGPS light illumination system and a UPlanSApo 4x objective (0.16 numerical aperture) was used. Each pixel corresponded to a 1.81x–1.91 µm square of tissue, and the field of view was 1440 × 1920 pixels. Image acquisition occurred at a frequency of 5–10 Hz.

### Analysis of Wide‐Field Imaging Data

The field of view was first pre‐defined by removing edges with no fluorescence signal to reduce data size. Subsequently, motion correction was performed by applying 2D normalized cross‐correlation^[^ ^89^ ^]^ between a reference frame (first frame) and the current frame. ROIs were then defined for excitation and inhibition, allowing us to convert them into time traces depicting changes in fluorescence (F) in response to stimulation compared to baseline. This was achieved using the mean intensity ± one standard deviation (SD) across all pixels within the pre‐defined field of view during 1s post‐stimulation period. To determine the location of peak intensity in response to stimulation, non‐responsive regions were first masked out. Then, the field was divided into subregions every 30 µm as a circular vector from the probe tip and averaged the pixel intensity within each subdivision.

### Analysis of Two‐Photon Imaging Data

Signal processing and data analysis were all handled using a customized MATLAB pipeline. As the initial standardized preprocessing of the images, CalmAn^[^ ^90^ ^]^ was used for motion correction, which utilizes NoRMCorre algorithm^[^ ^91^ ^]^ to correct non‐rigid motion artifacts. After the motion correction, neuronal soma regions of interest (ROIs) were manually extracted based on the presence of clear, morphologically distinct single neurons, utilizing the mean image across all recorded frames to increase signal‐to‐noise ratio.

Our images were from single‐plane recording; therefore, to suppress neuropil contamination from each soma due to possible signal spread from upper or lower planes, the neuropil area was defined as 1.5 times larger than the center of the soma ROI. Subsequently, this ring‐shaped neuropil fluorescence was subtracted using a weight of 0.7. Additionally, apart from somatic activity, neuropil activity was defined by subtracting all somatic ROIs from the imaged field of view, a method inspired by.^[^ ^92^ ^]^

### Simulation to Compare Electric Field Distribution Between Micro‐Electrode and Micro‐Coil

Numerical simulation was performed using COMSOL Multiphysics to compare µES and µMS, and a frequency domain analysis was applied at 200 Hz. A cylindrical space (diameter 500 µm, height 1 mm) was simulated, where both devices had a penetration depth of ≈200 µm, and the boundaries were set to a ground potential of 0 V. For µES, a current of 20 µA was applied, and the diameter of the device was set to be 3 µm, the electrical conductivity of metallic electrode was 4 × 10^6^ S m^−1^ (platinum‐iridium alloys). For the micro‐coil device, a voltage of 120 mV was applied to one terminal, and the other was grounded, with both terminals set to an infinite boundary condition. The voltage was adjusted to ensure that the integrated current inside the coil remained 100 mA, matching experimental measurements. And the model geometry was based on experimental design. The electrical conductivity of the aluminum coil was set to 3.7 × 10^7^ S m^−1^, while platinum‐iridium alloy was used for µES with an electrical conductivity of 4.0 × 10^6^ S m^−1^. The electrical conductivity of brain tissue was set to 0.25 S m^−1^ based on the previous study,^[^ ^93^ ^]^ and a low electrical conductivity of 1.0 × 10^−13^ S m^−1^ was used for parylene C material.

### Quantification and Statistical Testing

To determine responsive ROIs to µMS and µES, the cells were selected where the mean fluorescence at 1s or 2s post‐stimulation exceeded 1.5 times the SD of the baseline period (−3 to −1 s pre‐stimulation period). In cases where longer electrical stimulation durations (e.g., 900 ms) induced longer response latencies, the 2 s post‐stimulus period was averaged to quantify response amplitudes for population activity (*N* = 1 mouse). Cells from low and medium‐level stimulation intensity were used to directly compare somatic calcium response between µMS and µES. The maximum or minimum (as peak) response amplitude was obtained for comparison. The waveform slope for each significantly modulated cell was determined by linear fitting of the trial‐averaged signal from stimulation onset to the time it reached its amplitude peak. Neuropil comparison was performed by collapsing all low and medium stimulation intensity conditions and comparing during the 1‐s post‐stimulus period.

To compare response amplitudes between conditions and during dual‐probe stimulation (concurrent stimulation of µES and µMS) a paired *t*‐test with a p‐threshold of 0.05 was used. Chi‐square statistics were used to compare the significance of the proportion of cells modulated between stimulation conditions. The analysis was restricted to low and medium stimulation intensities to compare the significantly modulated cells between micro‐coil and micro‐electrode stimulation and neuropil activity.

### Ethical Statements

Laboratory animals were used and stated that the animal's care was in accordance with institutional guidelines.

## Conflict of Interest

The authors declare no conflict of interest.

## Author Contributions

K.K. and X.L. both contributed equally to this work. K.K. performed the experiment, analyzed data, and wrote the manuscript. X.L. produced the micro‐coil, performed the experiment, and wrote the manuscript. B.C. performed numerical simulations and wrote the manuscript. G.A.E.A. wrote the manuscript. S.G. produced the micro‐coil. L.G. and R.B. developed the viral vector. A.Y. performed the experiment. W.J.A. was responsible for conceptualization and wrote the manuscript. A.H. provided conceptualization, supervision, wrote the manuscript, and acquired funding. C.C. provided conceptualization, supervision, wrote the manuscript, and acquired funding. All of the authors participated in reviewing and editing the original draft.

## Supporting information



Supporting Information

## Data Availability

The data that support the findings of this study are available from the corresponding author upon reasonable request.
