# Supplementary material for: Micro‐Coil Neuromodulation at Single‐Cell and Circuit Levels for Inhibiting Natural Neuroactivity, Neutralizing Electric Neural Excitation, and Suppressing Seizures
Source: Adv Sci (Weinh). 2025 Apr 17;12(22):2416771. doi: 10.1002/advs.202416771 (PMC12165067; doi:10.1002/advs.202416771)
Supplement: Supplementary file 1 — Supporting Information [file ADVS-12-2416771-s001.docx]

**Supplementary Figures**


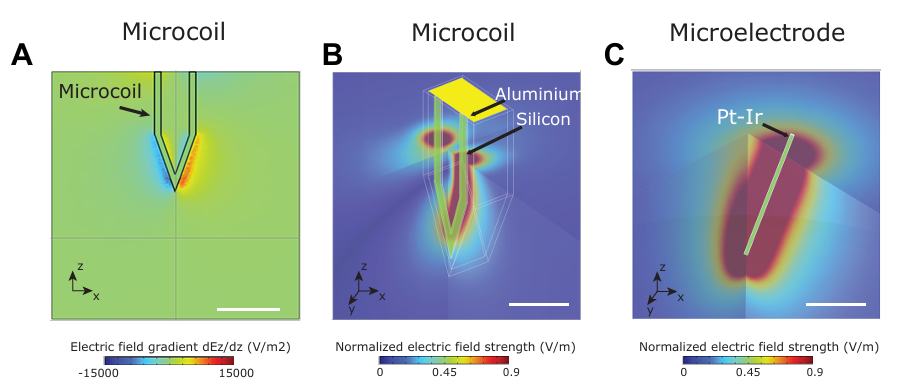


**Supplementary Figure 1.** Numerical simulation of induced electric field gradient by micro-coil (A). Normalized field strength distribution in 3D space by micro-coil (B) and microelectrode (C). Scale bar, 100µm

**
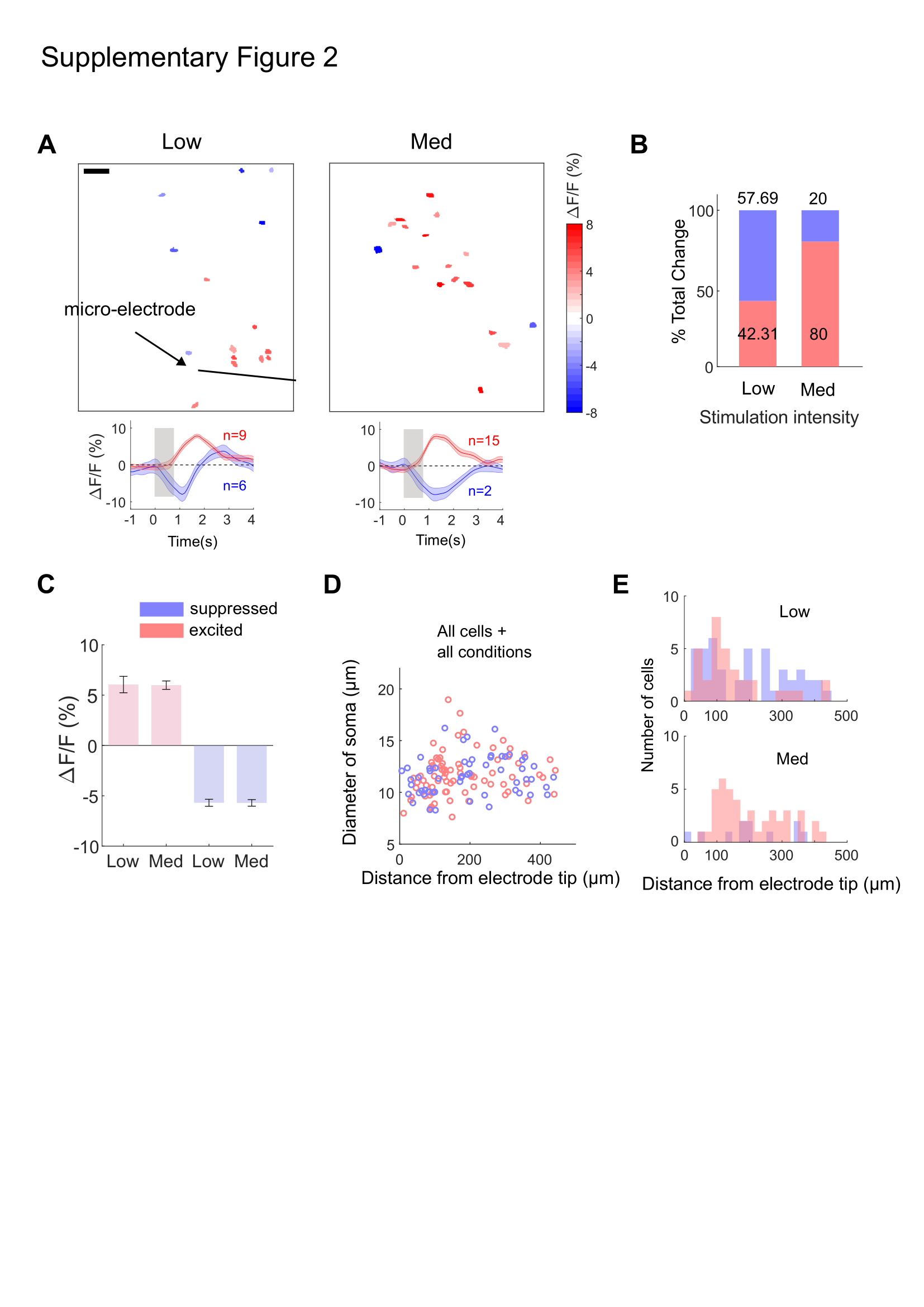
**

**Supplementary Figure 2.** TPM during micro-electrode stimulation. Same convention as main Figure2.
